# Cardiovascular and Metabolic Benefits of Extra Virgin Olive Oil Phenolic Compounds: Mechanistic Insights from In Vivo Studies

**DOI:** 10.3390/cells13181555

**Published:** 2024-09-16

**Authors:** Gabriele Serreli, Anna Boronat, Rafael De la Torre, Josè Rodriguez-Moratò, Monica Deiana

**Affiliations:** 1 Department of Biomedical Sciences, University of Cagliari, Cittadella Universitaria SS 554, 09042 Monserrato, Italy; 2 Department of Medicine and Life Sciences, Universitat Pompeu Fabra, 08003 Barcelona, Spain; 3 Integrative Pharmacology and Systems Neurosciences Research Group, Hospital del Mar Research Institute, 08003 Barcelona, Spain; 4 Physiopathology of Obesity and Nutrition Networking Biomedical Research Centre (CIBEROBN), 28029 Madrid, Spain

**Keywords:** cardiovascular diseases, cell signaling, clinical trials, extra virgin olive oil, metabolic diseases, polyphenols

## Abstract

Extra virgin olive oil (EVOO) represents a significant source of monounsaturated fatty acids (MUFA) and vitamin E, but it is also considered a functional food, due to the content of peculiar bioactive molecules, such as phenolic compounds, being able to modulate various processes related to aging and the most common metabolic and degenerative diseases. A lot of experimental research has focused on some of these components, but in most cases, the studies were performed in vitro testing compounds at non-physiological concentrations and achieving results that cannot easily be translated in vivo. Recent clinical studies demonstrated that in vivo these compounds are able to regulate physiological functions and prevent several pathological events including metabolic and cardiovascular diseases (CVDs), which represent the main causes of death worldwide. This review aims to sum up the major evidence on the beneficial effects of EVOO phenolic compounds in vivo on these pathologies, describing and evaluating the efficacy in relation to the mechanisms of diseases of the whole phenolic fraction and some of its specific components.

## 1. Introduction

Extra virgin olive oil (EVOO) is one of the cornerstones of the so-called traditional Mediterranean diet (MD) and is considered one of the most important foods for the supply of nutrients and substances with nutraceutical and health values [1]. Among the bioactive substances present in EVOO, monounsaturated fatty acids (MUFA) (>70%), α-tocopherol, and a small hydrophilic fraction (1–2%), including polyphenols, are the most valuable in terms of biological relevance [1,2]. Phenolic compounds have been widely studied in in vitro experiments for their powerful antioxidant activity, but it has also been observed that they are able to act as potent anti-inflammatory and antiproliferative agents, modulating several intracellular signaling pathways [3,4,5,6].

**Figure 1:**
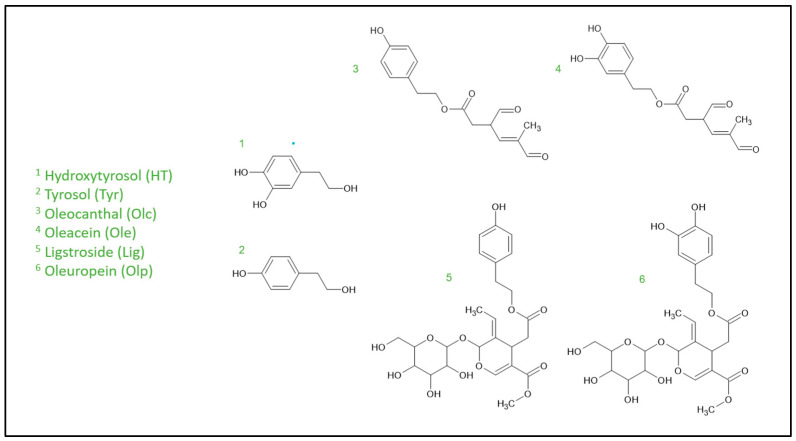
Chemical structures of main phenylethanoids and secoiridoids present in EVOO.

Among the most concentrated phenolic compounds present in EVOO, we find secoiridoids such as oleacein (Ole), oleuropein (Olp), oleocanthal (Olc) and ligstroside (Lig), but, above all, their phenylethanoid derivatives hydroxytyrosol (HT) and tyrosol (Tyr) [7] (Figure 1). Minor compounds such as flavonoids, phenolic acids, and hydroxyisochromans are also present, albeit in smaller concentrations [7,8].

The concentration of these compounds in EVOO is quite variable and depends on a series of factors including cultivar, the area of origin of the product, the fruit ripening stage, and production methods [1]. A study carried out in Italy comparing different cultivars from different geographical areas showed great variability while maintaining relative proportions of concentration among different compounds. Olp aglycon and its dialdehydic form were found to be by far the most concentrated (87.7–1546.61 mg/kg and 21.9–1682.89 mg/kg, respectively), followed by Lig aglycone (80.83–899.48 mg/kg). HT (1.30–53.41 mg/kg), Tyr (0.03–62.30 mg/kg), and other minor compounds such as luteolin (11.58–16.82 mg/kg), apigenin (1.05–2.91 mg/kg), and elenolic acid (1.41–30.75 mg/kg) were significantly less concentrated [9]. Another study on EVOO of Greek origin also measured the quantity of Ole and Olc, showing significant concentrations (183.9–355.0 mg/kg and 104.8–291.7 mg/kg, respectively) [10].

Most of the observed biological effects in vitro ascribed to EVOO phenolic compounds are not corroborated when tested in human clinical studies. Various factors may contribute to the unfruitful translation of the biological effects pointed out in vitro to human trials. Firstly, it has been observed that doses compatible with the human diet are hardly ever tested in in vitro investigations, but tested concentrations are actually often higher and not to a small extent. Secondly, in the specific case of EVOO containing simple phenols like HT, concentrations of the unchanged compound reaching the bloodstream are less than 1% of the ingested amount, with very low concentrations (a few nM) [11]. Conversely, HT and Tyr phase I/II metabolites can reach 50 to 100 times higher concentrations depending on the dose and other factors [12]. In this regard, in vitro studies have revealed that these metabolites display the same effects exerted by their parent compounds, often acting as modulators of cell signaling, and are thus likely responsible for the biological activities associated with EVOO simple phenols [13,14]. Thirdly, polymorphisms related to drug-metabolizing enzymes and transporters can lead to significant differences from individual to individual in the bioavailability of the phenolic compounds taken with EVOO, representing a further issue when translating scientific evidence obtained in vitro into in vivo studies [12]. Also, gender differences both in metabolic disposition and biological effects have to be considered [15].

Notwithstanding this, several clinical and animal studies have been carried out in the last twenty years demonstrating that part of the evidence obtained in vitro is likewise verified in vivo, albeit with different proportions. Some of these have focused on a single phenol, primarily HT, while others have evaluated the phenolic fraction as a whole. Since cardiovascular diseases (CVDs) are still the foremost cause of death in the world, associated with a global increase in the presence of predisposing factors including some chronic metabolic conditions (such as diabetes and hypertriglyceridemia) [16,17], several clinical trials have been carried out over the last 15 years to highlight the beneficial effects in the prevention of these pathologies deriving from the intake of EVOO and contextually of its phenolic fraction.

In the present review, our aim was to delve into the potential beneficial effects of EVOO phenolic compounds, in relation to possible mechanisms of their action, as candidates to be incorporated into nutraceutical formulations and as an active ingredient in functional foods, particularly in their preventive capacity against cardiovascular and metabolic diseases. This entails considering the use of larger (pharmacological) doses of the phenolic compounds, within a different context in terms of bioavailability and metabolism, as well as potential synergistic effects with other compounds in the nutraceutical formulation. Additionally, it is crucial to acknowledge that physiological responses may vary at higher doses, therefore raising pertinent safety concerns. The goal of this review is therefore to compile and describe the major proof of the biological efficacy of EVOO phenolic compounds derived from pre-clinical animal studies and clinical studies in the context of nutraceuticals and functional oils. Our goal is to provide a comprehensive estimation of the effects that are actually verifiable in vivo, especially in the context of cardiovascular and metabolic pathogenesis. Furthermore, the intracellular signaling pathways and gene expression involved in the effects verified, if present, will be highlighted to link macroscopic in vivo effects to what occurs at the cellular level.

## 2. Clinical and Animal Studies

### 2.1. Nutraceuticals Formulations Containing HT

HT is by far the most studied EVOO polyphenol due to its multiple biological functions [4]. In addition to the countless in vitro studies, which have highlighted all its nutraceutical and health benefits that go beyond its ability to scavenge free radicals, much research has been carried out on animals and clinical trials on humans, which we report in the following subheadings.

#### 2.1.1. Safety of HT Used as Novel Food

The European Food Safety Authority (EFSA) deems HT used as a novel compound for its use in functional foods and nutraceuticals as safe in humans under the proposed uses and levels (HT added to margarine up to 175 mg/kg and fish and vegetable oils up to 215 mg/kg). There are no reports regarding the possible genotoxicity of HT. A subchronic 90-day oral systemic toxicity study in rats testing HT at dose levels of 5, 50, or 500 mg/kg body weight (bw) per day, based on changes in body and organ weights in the highest dose group tested in this investigation, allowed to conclude that the dose of 50 mg/kg bw per day can be considered the no observed adverse effect level (NOAEL). Considering the NOAEL of 50 mg/kg bw per day in the HT subchronic oral toxicity study and the maximum anticipated daily intake for the HT as novel food, the margin of exposure (MoE, i.e., the ratio between the NOAEL and the maximum anticipated daily intake of HT) would result in doses of 100 mg/day for children (3–9 years of age) and at least 200 mg/day for adolescents, adults (excluding pregnant and breastfeeding women), and elderly people [18,19,20].

The Food and Drug Administration considers that a daily intake of HT at levels up to 51.06 mg/day is unlikely to cause any adverse effects and the compound is considered to be GRAS (Generally Recognized As Safe) [21].

#### 2.1.2. Animal Studies

HT has been studied in several animal models, mainly for the evaluation of its anti-inflammatory activity and prevention of CVDs. At the cardiovascular level, HT (10 mg/kg/day for ten weeks) showed harmful effects in increasing circulating monocytes expressing Mac-1, total cholesterol level, and atherosclerotic lesion areas in apoE-deficient male mice on a standard chow diet. The authors of this research suggested that phenolic-enriched nutraceuticals, out of their original matrix, may exert adverse consequences instead of beneficial effects [22], acting by decreasing post-transcriptional apolipoprotein A-I expression and, with a consequent oxidative mechanism, increasing the concentration of oxidized low-density lipoproteins (ox-LDL). More recently, the López de las Hazas’s group has also shown an increase in body weight in humanized mice with similar human lipoprotein metabolism after supplementation with HT (0.03% in a purified control diet), which also induced the accumulation of triacylglycerols in some tissues like mesenteric and epididymal white adipose tissues. In addition, systemic dyslipidemia after HT supplementation and impaired glucose metabolism were found, following modulation of the expression of genes related to inflammation and lipid metabolism [23]. Conversely, in healthy male Wistar rats, HT and its derivative HT–acetate (HT-Ac) compounds were administered once per day for one week and subsequently were shown to inhibit collagen-induced platelet aggregation in whole blood, with an ID50 of 48.25 mg/kg per day for HT and 16.05 mg/kg per day for HT-Ac. Among the mechanisms suggested by the authors for this biological event, we find the inhibition of platelet synthesis of thromboxane B2 (TxB2) (100 mg/kg, 30% from HT and 37% from HT-Ac) and the production of vascular prostacyclins (27.5% from HT and 32% from HT-Ac); in the same context, vascular nitric oxide (NO) release rose to 34.2% by HT and 66% by HT-Ac [24], thus contributing to the inhibition of platelet aggregation. Other HT-conjugated compounds (ethyl, butyl, hexyl, octyl, and dodecyl, 20–50 mg/kg/day) were given once per day for seven days and then the inhibition of TxB2, the plasma concentration of lipid peroxides and the platelet aggregation in male Wistar rats were observed. Interestingly, the administration of the butyl, hexyl, and octyl derivatives or the hexyl derivative modulated the thromboxane/prostacyclin ratio by counteracting thromboxane production, whilst longer-chain ethers preferentially inhibited prostacyclin production. Moreover, aortic NO metabolites and red blood cell glutathione (GSH) levels were also enhanced, thus including among the probable mechanisms involved an antioxidant action of the tested compounds and the activation of NO release, which acts as an anti-aggregating agent at the endothelial level [25].

Regarding metabolic diseases, HT (10 mg/kg/day by gavage for six weeks) was tested in a nutritional rat model of non-alcoholic fatty liver disease (NAFLD) and insulin resistance (IR) with a high-fat diet (HFD). HT-treated rats showed a noticeable decrease in serum cholesterol, aspartate aminotransferase (AST), and alanine aminotransferase (ALT), and increased insulin sensitivity and glucose tolerance. The HT effect was correlated to its ability to increase the expression of the gene related to hepatic peroxisome proliferator-activated receptor (PPAR)-α and its downstream-regulated gene fibroblast growth factor 21, the mRNA carnitine palmitoyltransferase 1A and the phosphorylation of acetyl-CoA carboxylase. Furthermore, it was also observed depletion of liver nitrosative/oxidative stress and inflammation decreased the nitrosylation of proteins, lipid peroxidation, and ROS production [26].

The ability of HT (10–50 mg/kg/day for seventeen weeks) to modulate the cellular redox state was also highlighted in a similar model developed in 4-week-old male C57BL/6J mice, where it ameliorated HFD-induced oxidative stress by improving antioxidant enzyme activities and normalized the expression of mitochondrial fission marker Drp1 and mitochondrial complex subunits. HT also significantly decreased serum lipids, fasting glucose, and the oxidation levels of proteins and lipids in both muscle and liver tissues in a db/db mice diabetic model. Furthermore, similar to the results in the HFD model, HT decreased muscle mitochondrial carbonyl protein levels and improved mitochondrial complex activities [27]. A synergistic effect was instead observed when administering HT and 3,4,-dyhydroxyphenylglycol (DHPG) (5 mg/kg/day + 1 mg/kg/day, respectively) in an experimental rat (2-month-old adult male Wistar rats) model of type 1 diabetes (T1D), where platelet aggregation, myeloperoxidase, oxidative and nitrosative stress, VCAM-1 and production of prostacyclin were significantly reduced after two months of daily administration [28].

#### 2.1.3. Human Clinical Trials

Regarding human clinical trials, HT has been studied mainly for its ability to limit the pathogenesis of atherosclerosis, as already observed in animal studies.

Quite recently, HT has been considered a useful supplement for the management of heart attacks in humans. In this study, a nutritional supplement containing 15 mg of HT/day was administered to patients 24 h after the onset of stroke for 45 consecutive days. Among the interesting results regarding the effects on the cardiovascular system, a decrease in the percentage of glycated hemoglobin and diastolic blood pressure, with mechanisms linked to the increase in the release of NO, was found, and the modulation of the expression of genes encoding for apolipoproteins, thus proving to be useful in the post-stroke recovery of patients [29].

In another recent study led by Cicero and colleagues [30], a single-arm, non-controlled, non-randomized prospective pilot clinical study recruited thirty hypercholesterolemic volunteers (aged 20–70 years, with LDL cholesterol (LDL-C) > 115 mg/dL and <190 mg/dL, an estimated 10-year atherosclerosis-related CVD risk <5% based on the SCORE (Systematic Coronary Risk Evaluation) risk charts) receiving dietary supplementation with highly standardized phenolic compounds (mainly HT) derived from Coratina olives (SelectSIEVE^®^ OptiChol). This intervention was associated with a significant regulation of systolic blood pressure, pulse pressure, LDL-C, high-density lipoprotein cholesterol (HDL-C), total cholesterol, fasting plasma glucose, and uric acid in comparison with baseline values. Still in patients presenting mild-to-moderate hypercholesterolemia (158 volunteers), a new food supplement called Body Lipid (BL), containing HT (5 mg), berberine (500 mg), coenzyme Q10 (2 mg) and monacolin K (10 mg), was shown to be successful in lowering LDL-C levels with a reduction of −26.3% [31]. A recent report on a randomized, double-blind, controlled crossover trial has instead shown how HT (3.3 mg) administered together with another phenolic compound from pomegranate, i.e., punicalagin (65 mg), significantly decreased the plasma levels of triglycerides and LDL-C in eighty-four (male and female) hypercholesterolemic subjects who received three capsules (HT + punicalagin + 331.7 mg of maltodextrin) daily for eight weeks [32]. One of the proposed mechanisms, based on previous studies [27], includes the modulation of cholesterol metabolism by decreasing the phosphorylation of MAPK p38 and, in turn, by the activation of AMP-activated protein kinase (AMPK) and inhibition of nuclear factor-kappa B (NF-κB). These mechanisms are known to trigger the upregulation of the LDL receptor (LDLR) and the blockade of sterol regulatory element-binding protein 2/proprotein convertase subtilisin/kexin type 9 (SREBP2/PCSK9). The same treatment was initially carried out by this research group to evaluate early atherosclerosis biomarkers in middle-aged, apparently healthy adults, although at 3-fold higher concentrations of HT (9.9 mg) and punicalagin (195 mg). It was observed that HT and punicalagin increased endothelial capacity in the endothelial dysfunction subgroup compared to placebo, and reduced oxidated LDL (ox-LDL) in subjects with higher levels of these oxidized lipoproteins. In addition, the prehypertension and hypertension subgroups treated with HT and punicalagin exhibited decreased diastolic and systolic blood pressure [33]. Still in the context of atherosclerosis prevention, a randomized double-blinded, placebo-controlled crossover trial was carried out to determine the effect of two gastro-resistant capsules containing HT (15 mg/day for three weeks) in forty healthy volunteers (18–65 years old). The analyses carried out in blood samples showed that total antioxidant status, oxidation biomarkers (thiols), and the gene expression of superoxide dismutase 1 (SOD1) were significantly increased, while malondialdehyde (MDA) and NO metabolites were decreased after HT administration [34].

Moreover, HT was given at a daily dosage of 45 mg for eight weeks to fourteen volunteers with mild hyperlipidemia to evaluate cardiovascular improvements and antioxidant effects. It was observed that the dosage of HT was safe but did not influence CVD biomarkers and inflammatory markers, electrolyte balance, blood lipids, and liver or kidney functions. However, HT demonstrated a physiologically relevant function by elevating endogenous vitamin C levels [35]. The only biological effect proposed by the authors to explain this event is merely that of an antioxidant action of HT, which may have limited the vitamin C turnover and depletion.

Moving on to metabolic diseases, a pioneering study led by Leger et al. [36] involved five T1D patients receiving 25 mg of HT the first day and 12.5 mg/day the following three days of the intervention. Interestingly, a 46% depletion of serum TXB2 after the fourth day was observed. Vitamin C slightly increased, but not in a statistically significant way, and plasma antioxidant capacity was improved after 1 h only.

Recently, a randomized double-blind placebo-controlled trial was performed to test the potential biological capacity, evaluated as improvement of oxidative stress parameters and liver ultrasound, of a mixture of 7.5 mg of HT and 10 mg of vitamin E in eighty adolescents with NAFLD. After a 4-month-long treatment, a decrease in oxidative stress biomarkers, triglyceride levels, insulin resistance, and steatosis grade was ascertained, and this advancement was found to correlate with the levels of carbonylated proteins and advanced glycation end products (AGE) [37]. Regarding the management of obesity and overweight, twenty-nine women received HT at two different administrations (5 and 15 mg/day) for six months. The intervention, deemed safe and well tolerated, led to important weight and visceral fat mass loss after four weeks. However, these effects were attenuated over time (12 and 24 weeks) [38]. Another study, again on overweight patients or others presenting type 2 diabetes (T2D), was conducted by including the consumption of bread enriched with HT in the diet, obtaining very promising results. In particular, sixty adults with overweight/obesity and T2D mellitus joined a 12-week dietary intervention based on the Mediterranean diet and consumed daily 60 g of conventional whole wheat bread or whole wheat bread enriched with HT. Those who consumed HT-enriched bread showed a greater fat mass decrease, and significant reductions were also reported in fasting glycated hemoglobin (HbA1c), glucose, and blood pressure in both groups after the intervention. However, the HT-enriched bread group showed greater decreases than the control group regarding glucose, blood lipid, insulin, TNF-α, adiponectin, and HbA1c [39].

As can be seen from these studies, there are differences in the approach and design of the investigation, with strengths and weaknesses. For instance, Fytili et al. [38] have investigated general health markers (body weight, fat mass, and metabolomics), which are useful for understanding broad health impacts but less clinically actionable compared to the specific metabolic outcomes found by Binou et al. [39] (HbA1c, lipids, and inflammation markers). Moreover, an older study like that led by Leger et al. [36] is very specific to thromboxane and CVD risk, making it a more niche but mechanistically insightful study. We also found different types of intervention: Fytili and colleagues have used HT as a supplement, while Binou et al. investigated its role as a functional food ingredient (wheat bread). This distinction could affect compliance and real-world application. Leger et al. used a natural extract from OO wastewater instead, exploring a sustainable and possibly cheaper alternative. The strength of the first of these three studies lies in its methodological rigor (randomized double-blind) showing the strongest design in terms of reducing bias. Moreover, it displays a broad focus on long-term health outcomes using metabolomics, ensuring high reliability in its findings. However, its lack of specificity in terms of health conditions could be seen as a limitation. The second of these studies taken as examples stands out for its clinical relevance, especially for people with T2D. The inclusion of multiple health markers makes it more actionable in healthcare settings, but the intervention (HT-enriched bread) may limit generalizability, while the third is a focused investigation of the HT effect on thromboxane, providing a clear mechanistic insight for T1D patients, but its narrow focus limits its broader applicability.

Another limitation of these investigations is the co-presence of compounds that contribute to the efficacy of the treatments, thus affecting the overall evaluation of the effects of HT in vivo. A proper example is the controlled study carried out by D’Addato et al. [31], where monacolin K was administered in addition to HT. Since monacolin K is an analog of lovastatin (a drug that has been used for decades to lower LDL), we can assume that the main effect is mainly due to the presence of this compound rather than HT (Table 1).

### 2.2. Nutraceuticals Formulations Containing Other EVOO Polyphenols

#### 2.2.1. Animal Studies

Among the other phenolic compounds studied in vivo for their biological properties, Olp is certainly the most popular. Olp, a secoiridoid compound incorporating a molecule of HT within its structure, has been shown to display numerous health-beneficial properties, evaluated across various animal models. As already seen for HT, Olp has been studied for its ability to preserve cardiovascular function through different mechanisms. A first study led by Coni and colleagues demonstrated that the dietary administration of 7 mg/kg of Olp increased the capacity of LDL to avoid oxidation and decreased the plasmatic amounts of total, free, and esterified cholesterol in twenty-four female New Zealand white rabbits [40]. Some years later, the efficacy of Olp (10 and 20 mg/kg daily for 6 or 3 weeks, respectively) on oxidative damage, infarct size, and the metabolic profile in hypercholesterolemic ischemic rabbits was evaluated. The concentrations tested were able to reduce infarct size in normal rabbits, while only the concentration of 20 mg/kg was effective in the hypercholesterolemic ones. Moreover, Olp administration decreased the plasmatic concentrations of protein carbonyl and lipid peroxidation products with respect to the untreated groups. In contrast, these factors rose relative to baseline in the control groups due to ischemia and reperfusion. Treatment with both doses of Olp for 6 weeks also reduced triglycerides and total cholesterol concentrations in plasma, acting primarily as an antioxidant substance [41]. The same research group tested Olp (20 mg/kg daily for 3 and 6 weeks) in male New Zealand white rabbits preconditioned with ischemia/reperfusion, and they found that the phosphorylation of Akt, PI3K, endothelial NO synthase (eNOS), STAT3 and AMPK in cardiac muscle cells were significantly higher in the preconditioning group and all Olp-treated groups (Olp alone or in co-treatment with cholesterol) compared to the control and the group treated with cholesterol [42]. Therefore, Olp mitigated the adverse effects of ischemia and hypercholesterolemia. Afterwards, Olp effects were studied on experimental autoimmune myocarditis (EAM) in 8-week-old male Lewis rats. EAM was induced by foot pads subcutaneous injections of 0.1 mL of porcine cardiac myosin (10 mg/mL) mixed with 0.1 mL Freund’s complete adjuvant (FCA) supplemented with mycobacterium tuberculosis H37Ra, and, after four weeks from first immunization, Olp (20 mg/kg/day) was then administered for the subsequent four weeks. Olp treatment has been shown to limit left ventricular end-systolic diameters, left ventricular end-diastolic diameters, and left ventricular end-diastolic pressures and to increase ejection fractions in post-myocarditis rats. Among the hypothesized mechanisms underlying these biological effects, the down-regulation of myocardial levels of p-IκBα, IKKα, and NF-κB p65 was observed. In addition, Olp inhibited CD4+, CD8+, and macrophage infiltration in the myocardium, and decreased the serum release of interleukin-1β (IL-1β), IL-6, and TNF-α [43]. An interesting investigation by Jin et al. reports the protective effect of Olp (20 mg/kg daily for 2 days) in the prevention counter to myocardial ischemia/reperfusion in rats. Specifically, Olp inhibited CK MB and LDH serum levels, myocardial infarction size, and attenuated phosphorylated extracellular signal-regulated protein kinase (ERK), phosphorylated mitogen-activated protein kinase kinase (MEK), p53, caspase 3, and p-IκBα activation. In addition, the Olp treatment decreased IL-1β, IL-6, TNF α, and MDA, and, conversely, increased GSH, SOD, and catalase levels [44].

Olp has also been extensively studied in animals for its effects on metabolic diseases such as obesity and diabetes. In this regard, research led by al-Azzawie and colleagues aimed to investigate the effects of Olp administration in reducing hyperglycemia and oxidative stress in alloxan-induced (intravenous dose of 150 mg/kg) diabetic male New Zealand rabbits. Following 16 weeks of Olp treatment (20 mg/kg daily), the blood levels of MDA, glucose, and most of the enzymatic and non-enzymatic and antioxidants (GPx, SOD) were significantly improved to values similar to those of normal control rabbits [45]. Herein, the antioxidative effect of Olp may have prevented further peroxidation and glycosylation of proteins by depleting ROS and, consequently, limiting their deleterious effects. Moreover, the authors speculated that Olp may have induced protein synthesis of SOD and GPx enzymes, thus explaining their observed high activity after the intervention. More recently, an Olp-rich diet was evaluated against T2D in Tsumura Suzuki Obese Diabetes (TSOD) mice. The supplementation (35% *w*/*w* Olp) lowered impaired glucose tolerance and hyperglycemia in diabetic mice from 10 to 24 weeks of age, but it showed no effects on obesity. In addition, it mildly reduced oxidative stress, measured as hydroxyoctadecadienoic acid content in plasma, by 26.2% [46]. The improvement effects of Olp on blood glucose level were not due to the amelioration of insulin secretion but to the decrease in IR, since plasma insulin levels were unchanged between untreated and Olp-treated mice. However, the biological processes by which IR was improved have not been clarified. Effects against obesity were instead observed in HFD C57BL/6N mice and, in particular, a significant reduction in HFD-induced body weight gain and visceral adiposity. Olp (0.03% *w*/*w* for 10 weeks) also significantly inhibited the HFD-induced increases of adipogenic-related gene expression involved in WNT10b- and galanin-mediated signaling in adipose tissue [47]. Similarly to Olp, the secoiridoid Ole was able to prevent the increase in adipocyte size and reduce the inflammatory infiltration of lymphocytes and macrophages in adipose tissue in HFD-fed mice. These biological evidences were associated with the modulation of the expression of adipose tissue-specific regulatory elements such as adiponectin, FAS, PPARγ sterol regulatory element-binding transcription factor-1 (SREBP-1), and insulin-sensitive muscle/fat glucose transporter GLUT-4 [48].

In addition, Lombardo et al. showed that Ole (20 mg/kg for five weeks) was able to modulate plasma glucose, cholesterol, and triglyceride serum levels in HFD-fed mice in comparison with normocaloric diet-treated mice. Furthermore, protein levels of FAS and SREBP-1 were modulated, confirming what was observed above [49]. More recently, an Ole-enriched diet (0.01% *w*/*w* for 24 weeks) was also able to normalize eNOS and nicotinamide adenine dinucleotide phosphate (NADPH) oxidase-1 overexpression in BALB/c mice injected with pristane, showing interesting properties against endothelial dysfunction [50]. Regarding Tyr, it was recently shown that it may act as a ligand to bind PPARα, activating the transcription of downstream genes and leading to lowered final body weight and hepatic lipid accumulation in male C57BL/6J HFD-fed mice supplemented with 0.025% (*w*/*w*) Tyr for 16 weeks [51].

In the context of cardiometabolic diseases, it must be taken into account that, as seen for HT, there may also be non-negligible adverse effects when taking these compounds, especially at high concentrations. For example, as shown by Sun and colleagues, Olp (60 mg/kg/day for eight weeks) is able to significantly lower blood pressure by acting at the hypothalamic level and modulating signals such as Nrf-2 and related genes, as seen in spontaneously hypertensive rats (SHR) [52], so, for this reason, the authors suggest that Olp may aggravate low blood pressure in humans who already have low blood pressure, and could also interact with other drugs that are selected to lower blood pressure or regulate diabetes [53].

#### 2.2.2. Human Clinical Trials

The phenolic compounds of EVOO, other than HT, have been little studied in humans in the context of nutraceutical studies. A pioneering study from Visioli et al. aimed to evaluate the antioxidant capacity of HT and Olp aglycone administered through an olive oil (OO) added with OO extracts owning increasing amounts of phenolics. The administration of phenol-rich OO to six healthy male volunteers was associated with a decreased urinary excretion of 8-iso-PGF2a, a biomarker of oxidative stress, and this effect was dose-dependent. In addition, a negative correlation between homovanillyl alcohol (HVAlc), the main methylated metabolite of HT, and F2-isoprostanes excretion was found, suggesting that this derivative may better reflect the in vivo biological effects of HT [54].

Research carried out by our group involving thirty-three patients (aged 50 to 80) with at least three CVD risk factors showed a few years later how another EVOO phenolic compound, namely Tyr, administered in capsules, together with white wine (1.4 mg or 2.8 mg for female and male patients, respectively), affected ratios of three circulating ceramide (Cer), biomarkers associated with an amelioration of endothelial function when compared to the control levels. Moreover, the intervention with Tyr + white wine was able to limit the alterations in plasma diacylglycerol levels observed after the administration of white wine alone [55]. It should be noted that the mechanisms underlying these biological effects have not been described. Moreover, it should be emphasized that Tyr probably was biologically converted to a more powerful compound such as HT. Indeed, the simultaneous administration of Tyr with ethanol has been reported to lead to the endogenous formation of HT, which could act in its place and bring benefits to the cardiovascular system [56]. This conversion of Tyr into HT is mediated by two isoforms of cytochrome P450 (CYP2D6 and CYP2A6) [56,57].

An olive leaf extract containing a mix of Olp and HT (51.1 mg Olp, 9.7 mg HT per day) was tested on CVD risk factors and insulin action in forty-six middle-aged overweight men in a randomized, double-blinded, placebo-controlled crossover study. This intervention was associated with a 15% increase in insulin sensitivity compared to the placebo, and there was also a 28% improvement in pancreatic β-cell responsiveness. In addition, HT and Olp supplementation also resulted in an increase in fasting IGFBP-1, IL-6, and IGFBP-2 concentrations. However, no effects on body composition, blood pressure, lipid profile, TNF-α, IL-8, ultra-sensitive CRP, carotid intima-media thickness, and liver function were observed [58]. More recently, EVOO with a high Olc amount was administered to 23 subjects presenting with metabolic syndrome and hepatic steatosis (fifteen men and eight women, age: 60 ± 11 years) daily for two months. This intervention was associated with a reduction in waist circumference, body weight, body mass index (BMI), and alanine transaminase, as well as TNF-α, IL-6, IL-17A, and IL-1β expression, while the IL-10 level was enhanced. However, it must be noted that a correlation between the presence of Olc in high concentrations and the observed effects was not statistically assessed [59]. Olc was also studied as a postprandial antiplatelet agent in T2D patients when administered with OO (40 mL OO with 500 mg/Kg Olc) in comparison with OO with low phenolic content (40 mL OO < 10 mg/Kg) or butter [60]. Moreover, similar effects were observed in healthy men who consumed 40 mL/EVOO, with different proportions of Olc, Ole, and Tyr. It was seen, with a blood sample taken after two hours, that collagen-stimulated platelet aggregation was inhibited after consumption of EVOO rich in Olc, while enrichment with Ole and Tyr did not lead to significant changes [61]. Finally, a randomized, controlled crossover study called APRIL (Aove in Prediabetes) aimed to evaluate the positive effects of Ole and Olc against T2D and obesity.

Briefly, people aged 40–65 years with obesity and prediabetes were subjected to an intervention consisting of substituting for 1 month the oil used for food, both raw and cooked, with EVOO rich in Ole and Olc or simple OO. Among the main outcomes, a decrease in weight, BMI, and blood glucose after treatment with EVOO in comparison with OO was found, as well as an increase in antioxidant defenses [62].

As seen for the studies on HT, and also in this case, there are many differences and limitations between the different trials. Studies like [58,59,62] focused on populations at risk for metabolic disorders: overweight middle-aged men, people with metabolic syndrome, and those with obesity/prediabetes, respectively. These are highly relevant groups for exploring the metabolic effects of EVOO polyphenols. The second of these studies provided the broadest assessment of multiple metabolic markers, including weight, liver function, and inflammation, making it the most comprehensive, with a 2-month intervention standing out for its slightly longer intervention period compared to the others. The recent study led by Ruiz-Garcia and colleagues assessed both inflammation and antioxidant status, offering insights into how Olc and Olc affect oxidative stress, but it lacks broader clinical outcomes. This is a crossover trial, showing a strong design, but the relatively short intervention periods limit the ability to detect longer-term effects. The study led by Katsa et al. [60] is more focused on T2D, specifically examining cardiovascular risks through platelet function, which is more specialized but crucial for diabetes management. This latest study is very narrow, examining only acute antiplatelet effects post-meal, providing mechanistic insights. Moreover, while acute effects are useful for understanding immediate responses, they may not provide insight into the long-term cardiovascular benefits of Olc consumption. Therefore, longer-term studies with a larger number of participants will be needed to confirm the clinical relevance of the results.

An aspect shared by these studies is the delivery of the compounds through enriched OO or OO highly concentrated in these substances. On the one hand, this administration simulates the conditions under which these compounds are commonly taken with the diet and then absorbed by our tissues; therefore, it should be deemed a strength of these trials. On the other hand, it is not possible to determine whether the obtained health outcomes are the result of an actual action of these compounds or the contribution of other compounds present in these OOs, including vitamins and polyunsaturated fatty acids (Table 2).

### 2.3. Studies Using Functional Oils Enriched with EVOO Phenolic Fraction

#### Clinical Studies

Given the paucity of animal studies regarding the phenolic fractions of EVOO, we have focused only on human clinical studies of biological interest. Some studies are derived from larger projects, such as PREDIMED (targeting primary cardiovascular disease prevention in subjects at high cardiovascular risk) and PREDIMED-Plus (targeting primary prevention of cardiovascular diseases in subjects with metabolic syndrome), and very often concern the effects of EVOO polyphenols on diseases of the cardiovascular system and metabolic diseases such as diabetes. The PREDIMED trial, perhaps the most important of these studies due to the relevance of its results (rediscussed after a revision in 2018), showed how the intake of EVOO and its hydrophilic fraction can reduce the risk of pathological events of a cardiovascular nature [63]. However, in the context of this review, it is right to point out that the benefit of EVOO consumption in the PREDIMED investigation was likely due to several factors, especially high-unsaturated-fat content together with micronutrients and phenolic compounds. Therefore, as suggested by Billingsley and Carbone [64], it is impossible to discriminate which factor has brought the most benefit, as probably the benefit is the result of the sum of all these beneficial elements. For this reason, we have not covered the results of this project in detail (which has already been performed extensively elsewhere) and we focused on the studies where the effects led by polyphenols are more evident.

The EUROLIVE (Effect of Olive Oil Consumption on Oxidative Damage in European Populations) study, developed in five European countries some years ago, helped to establish how OO phenols are effective in limiting LDL oxidation in plasma. In particular, it was seen that 25 mL/day of 3 OOs with low- (2.7 mg/kg), medium- (164 mg/kg), and high- (366 mg/kg) polyphenol content were able to modulate LDL oxidation in a dose-dependent manner in healthy volunteers in an intervention period of three weeks. Results also showed a significant decrease in ADRB2, IL8RA, ORL1, and CD40 gene expression with the decrease in LDL oxidation and a significant decrease in intercellular adhesion molecule (ICAM)-1 with increasing amounts of Tyr and HT in urine. Moreover, a direct relationship was observed between ox-LDL autoantibodies (OLAB) and the total OO phenolic content in LDL. OLAB concentrations, adjusted for ox-LDL, increased directly in a dose-dependent fashion with the phenolic content of the OO administered. Interestingly, the plasma apo B-100 levels and the number of small and total LDL particles also decreased from baseline after the intervention [65,66,67,68]. These results confirmed and added new information to what had been found by the same research group in a similar study carried out a few years earlier, where for the first time the degree of LDL oxidation was found to be lessened as the phenolic content of the OO administered increased [69].

Another intriguing double-blind, randomized, controlled, crossover study, the Australian OLIVAUS, sought to elucidate the potential effect of consuming EVOO with high-polyphenol content regarding blood pressure and the modulation of plasmatic lipoprotein concentrations. Fifty healthy volunteers consumed 60 mL/day of either low-polyphenol EVOO (86 mg/kg polyphenols) or high-polyphenol EVOO (360 mg/kg polyphenols) for three weeks. Anthropometric data, arterial stiffness, peripheral blood pressure, and central blood pressure were assessed at baseline and follow-up, as well as serum HDL-cholesterol efflux capacity, total antioxidant capacity (TAC), plasmatic ox-LDL, anthropometrics, and circulating lipids and high-sensitivity C-reactive protein (hs-CRP). Notably, it was noticed a significant decrease in central and peripheral systolic blood pressure after high-polyphenol EVOO consumption. Moreover, plasma ox-LDL decreased, and TAC increased only in the high-polyphenol EVOO consumption. Stratified analyses were also performed by CVD risk status defined by inflammation or abdominal obesity. In the abdominal obesity group, ox-LDL levels were lowered, and TAC increased only after high-polyphenol EVOO consumption. In addition, in the subgroup with inflammation, hs-CRP levels were decreased, again only in the high-polyphenol EVOO-treated group, demonstrating how the concentration of polyphenols in EVOO is decisive for the effects observed at the cardiovascular level after its intake [70,71,72,73].

In another recent study, precisely a randomized, controlled, parallel-arm, clinical study with forty patients with at least one classic CVD risk factor who were referred to coronary angiography were randomly assigned to two groups and received 25 mL low-polyphenol refined OO (ROO) or high-polyphenol EVOO daily for six weeks. Plasma CRP and LDL-C were significantly reduced in patients who received EVOO, confirming what has already been seen above [74]. In the same context, in a randomized, controlled, crossover trial, thirteen pre-/hypertensive patients received 30 mL of two similar OOs with moderate- (289 mg/kg) or high- (961 mg/kg) polyphenol content. After high-polyphenol OO intake, with respect to the moderate one, a postprandial increase in PPARα, PPARγ, PPARδ, (PPAR)BP, scavenger receptor class B type 1, ATP-binding cassette transporter-A1, and CD36 gene expression in macrophages it was observed, thus indicating a prominent role of OO polyphenols in the upregulation of genes involved in the cholesterol efflux from these cells to HDL [75].

A similar study was carried out in twenty-four young women with high-normal blood pressure or stage 1 essential hypertension, through a double-blind, randomized, crossover dietary intervention trial. When compared to baseline levels (run-in period of four months), only the polyphenol-rich OO diet (two months intervention) led to a significant drop in systolic and diastolic blood pressure, associated with a reduction in plasma CRP, ox-LDL, and serum asymmetric dimethylarginine (ADMA). In addition, the polyphenol-rich OO diet also promoted an increase in plasma NO metabolites and hyperemic area after ischemia [76]. Similarly, Valls et al. showed an improved endothelial function, assessed as ischemic reactive hyperemia, and a decrease in plasmatic ox-LDL after the intake of EVOO with a high content of polyphenols compared to that with low content [77].

Altogether, these results suggest a strong participation of polyphenols in the maintenance of cardiovascular function and blood pressure and in the perspective of preventing diseases such as atherosclerosis. In this regard, it was also recently noted that a higher intake of EVOO phenols, including HT and Tyr, was associated with a lower risk of positive coronary calcium in a trial performed on 2318 men to evaluate the presence of plaques in femoral and carotid arteries and coronary calcium [78]. An older randomized crossover and blind study on fourteen healthy and fourteen hypertriacylglycerolaemic patients investigated whether EVOO polyphenols decrease postprandial levels of soluble isoforms of ICAM-1 and vascular CAM (sVCAM)-1, after a high-fat meal. The study involved a one-week adaptation to a diet supplemented with EVOO containing 1.125 mg polyphenols/kg and 350 mg tocopherols/kg, or ROO without polyphenols or tocopherols. Blood samples taken over the following 8 h demonstrated a similar postprandial triacylglycerol response for both ROO and EVOO meals. However, in both hypertriacylglycerolaemic and healthy patients, the incremental area under the curve for sVCAM-1 and sICAM-1 significantly decreased after the EVOO intervention when compared to the ROO group [79]. Among the upstream effects suggested by the authors but not actually verified in the study, there could be an increase in the bioavailability of NO and the suppression of NF-κB expression. Other results from a randomized, sequential crossover design regarding twenty-one hypercholesterolemic volunteers that received two breakfasts rich in OOs with different phenolic contents (80 or 400 ppm), showed a higher decrease in plasminogen activator inhibitor-1 PAI-1 activity in plasma 2 h after the high-polyphenol meal than after the low-polyphenol meal [80]. The presence of polyphenols in EVOO at high levels has also proved to be responsible for the down-regulation of proatherogenic and pro-inflammatory genes (*ARHGAP15*, *POLK*, *IFNγ*) in peripheral blood mononuclear cells, also decreasing plasma inflammatory and oxidative markers and the expression of related genes [81].

In this context, it is worth mentioning a systematic review and meta-analysis proposed by George et al. to summarize the main evidence obtained from these human studies. Randomized, controlled trials (twenty-six studies) that investigated markers of CVD risk were included, and a meta-analysis was conducted using clinical trial data with available CVD risk outcomes. Compared to low-polyphenol OO, high-polyphenol OO significantly decreased MDA, ox-LDL, and total cholesterol, and improved HDL cholesterol. In addition, subgroup and individual studies reported further improvements in inflammatory markers and blood pressure [82]. As anti-inflammatory compounds, the phenolic fraction of EVOO was evaluated at the cardiovascular level in the study called NUTRAOLEUM [83,84], in which fifty-three healthy subjects consumed EVOOs with a high-polyphenols and triterpenes content and this led to a plasmatic reduction in oxidized lipids, inflammatory biomarkers such as TNF-α and other cytokines, which are indicators of DNA damage and vascular damage.

As already observed for the single compounds, the entire phenolic fraction has also been evaluated against T2D in clinical studies. A randomized, controlled, double-blind, crossover trial of twenty adults at risk for T2D (presenting metabolic syndrome or prediabetes) assigned 50 mL of high-polyphenolic EVOO or 50 mL of ROO without polyphenols. Improvements in endothelial function assessed as flow-mediated dilatation were observed in the high-polyphenolic EVOO group compared to the ROO, while no significant effects on diastolic or systolic blood pressure were detected [85]. Furthermore, high-polyphenolic EVOO (25 mL/day, 577 mg of polyphenols/kg) significantly lowered fasting plasma HbA1c and glucose levels, as well as BMI and body weight in eleven overweight patients with full-blown T2D. Also, high-polyphenolic EVOO intake provoked a reduction in serum levels of ALT, AST, and visfatin [86].

Taking into consideration the main macro-studies highlighted in this section, namely EUROLIVE and OLIVAUS, we find in some cases a complementarity of the results obtained, but there are also limitations and useful indications for future research. The EUROLIVE study focused on healthy individuals from Europe, whereas the OLIVAUS study targeted Australian adults at risk for cardiovascular disease. As a result, the outcomes of OLIVAUS are more relevant for individuals who are likely to develop CVD conditions or have metabolic risk factors. In addition, the inclusion of an at-risk population in OLIVAUS makes the findings more applicable to clinical settings, whereas EUROLIVE’s results are more useful for general health promotion in healthy populations. The EUROLIVE study was relatively short at 3 weeks, while OLIVAUS lasted 3 months. The longer duration of OLIVAUS provided stronger evidence for sustained health benefits, especially in terms of inflammation and cholesterol management. However, EUROLIVE’s shorter duration was still sufficient to detect changes in oxidative stress and HDL cholesterol, although it limits the understanding of longer-term effects. Furthermore, both studies showed positive effects on HDL cholesterol and oxidative stress. However, the OLIVAUS study also demonstrated a reduction in LDL-C and inflammation, which are critical risk factors for CVD, providing a more comprehensive assessment of OO cardiovascular benefits. Regarding the interventions, EUROLIVE compared OO with varying polyphenol concentrations, showing a dose–response relationship for oxidative stress reduction and HDL improvement. OLIVAUS used a high-polyphenol EVOO and found additional benefits for inflammation and LDL reduction, emphasizing the broader health impact of high-polyphenol OO. In the EUROLIVE study, a smaller daily dose of 25 mL of OO was administered, while in the OLIVAUS study the administered dose was 60 mL/day. The higher dose of OLIVAUS may have contributed to the more robust changes in inflammation and LDL-C. This suggests that a higher intake of EVOO, similar to traditional MD, may be necessary for maximum cardiovascular benefits (Table 3).

## 3. Concluding Remarks

EVOO phenolic compounds are safe in a large range of doses and are categorized as GRAS compounds for humans. Consequently, animal studies and clinical trials examining the beneficial effects of EVOO phenols have increased in the last twenty years and enlarged our understanding of the mechanisms of their action in the prevention of chronic degenerative pathologies. The updated evidence underscores the potential of phenolic compounds found in EVOO as nutraceuticals or active ingredients in functional foods. These compounds demonstrate the ability to preserve cardiovascular function and protect from metabolic diseases through various mechanisms.

**Figure 2:**
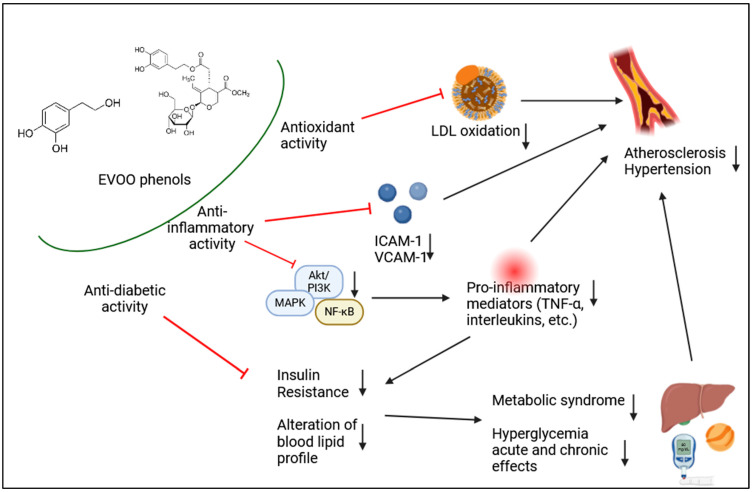
Main mechanisms involved in the protective effects of EVOO phenolic compounds against cardiovascular and metabolic diseases.

Current findings on the in vivo efficacy of EVOO phenolic compounds reveal an undoubtedly positive impact on several markers of inflammation and oxidative stress in a dose-dependent fashion. Furthermore, they are able to modulate the gene expression of several antioxidant enzymes, showing interesting insights and confirming what has been observed in nutrigenomic studies [87]. Thanks to their antioxidants and anti-inflammatory properties, well documented in vitro, the whole phenolic fraction as well as the most active pure phenolics have been shown to modulate in vivo important pathways involved in the pathogenesis of CVD and metabolic diseases (Figure 2). A specific health effect has been shown at the vascular level, where EVOO polyphenols show an efficacious anti-atherogenic action, through the maintenance of vascular function and blood pressure, and the inhibition of the endothelium adhesiveness (i.e., lowering adhesion molecules synthesis and exposure) and thrombogenicity. Other than acting as antioxidants, the mechanisms by which phenolic compounds exhibit these beneficial properties are deemed to include their interaction with PI3 kinase/Akt and MAPK signaling cascades and related activation of transcription factors, which regulate cell function and the release of pro-inflammatory factors. In vivo studies have also highlighted the ability of EVOO polyphenols to regulate glucose and lipid metabolism and increase insulin sensitivity, through molecular mechanisms not yet clarified, but probably linked to the modulation of gene expression like those encoding lipoproteins and their major components. The modulation of plasma lipids profile and blood sugar level is the key factor in the beneficial effects observed in the prevention of metabolic illnesses, such as T2D.

All the mentioned reasons make HT and other less studied EVOO phenolic compounds excellent candidates for their application as a nutraceutical or functional ingredient. Nonetheless, several complex issues, raised exactly by the in vivo studies, still remain unresolved and need to be further investigated to establish the ideal dose to be used in a defined physio-pathological context and the food matrix for maximum bioavailability and thus biological activity. Bioavailability, however, must be referred to the whole complex mixture of parent compounds and metabolites originated by in vivo metabolic activity and biotransformation, which are important contributors to the discrepancies between in vitro and in vivo studies and observed in vivo interindividual variability in the biological effectiveness of EVOO phenolic compounds.

Future research avenues will be crucial for strengthening the current understanding of the health benefits associated with EVOO phenolic compounds. Conducting larger human clinical trials would not only provide more robust data but also help address existing variability in smaller studies, ensuring more reliable conclusions. Additionally, long-term studies will be necessary to assess the sustainability and potential cumulative effects of these compounds over extended periods, thus offering insights into their real-world applicability.

Moreover, exploring the clinical applications of EVOO phenolics could open new pathways for developing preventive or therapeutic interventions for various chronic conditions, including CVD, neurodegenerative disorders, and inflammation-related conditions. Integrating this research into dietary guidelines would also allow healthcare professionals to provide evidence-based recommendations to patients, improving public health outcomes by encouraging the consumption of functional foods rich in beneficial bioactive compounds. For these reasons, more in vivo research is still needed to correctly understand and predict the effects of EVOO polyphenols intake, in view to design tailored dietary interventions to improve health.

## Figures and Tables

**Table 1:** HT tested in animal models and human clinical studies against cardiovascular and metabolic diseases.

Type of Intervention	Concentration Tested	Effects	References
Animal models			
Homozygous apoE KO mice	10 mg/kg/day for ten weeks	Harmful effects by decreasing apolipoprotein A-I and increasing atherosclerotic lesion areas circulating monocytes expressing Mac-1 and total cholesterol	[22]
Human APOB100 transgenic (HAPOB100Tg) male mice on the C57BL/6 background	Purified control diet (*n* = 8) without or with HT (0.03%) for 8 weeks	Induced systemic dyslipidemia and impaired glucose metabolism. Accumulation of triacylglycerols in mesenteric and epididymal white adipose tissues	[23]
Healthy male Wistar rats	1, 5, 10, 20, 50, and 100 mg/kg/day for seven days.	No toxicity observed and inhibition of platelet aggregation (ID50 48.25 mg/kg) and of platelet synthesis of TxB2. Increase in NO release	[24]
Healthy male Wistar rats	HT esterified derivatives, 20 mg/kg/day for seven days	Inhibition of platelet aggregation, TxB2, and plasma concentration of lipid peroxides. Increase in nitrites and GSH in red blood cells	[25]
Nutritional rat model of insulin resistance (IR) and NAFLD by HFD	10 mg/kg/day for five weeks	Reduction in serum cholesterol, AST, and ALT. Improvement of insulin sensitivity and glucose tolerance. Increase in hepatic PPAR-α. Reduction in liver inflammation and the nitrosylation of proteins, ROS release, and lipid peroxidation	[26]
HFD in C57BL/6J mice	10 mg/kg/day for seventeen weeks	Counteraction of oxidative stress by inducing antioxidant enzyme activities, and modulation of the expression of mitochondrial fission marker Drp1 and mitochondrial complex subunits	[27]
C57BL/6J mice db/db diabetic model	10 mg/kg/day for seventeen weeks	Decrease in fasting glucose, serum lipids, and the oxidation levels of proteins and lipids	[27]
Experimental adult male Wistar rats model of T1D	HT 0.5 mg/kg/day for two months	Reduction in platelet aggregation, myeloperoxidase, oxidative and nitrosative stress, production of prostacyclin, and VCAM-1	[28]
Clinical studies			
Randomized, controlled, double-blind pilot study involving eight individuals with minor or moderately severe acute ischemic stroke	Treatment with a daily nutritional supplement containing 15 mg of HT (or placebo for 45 days)	Decrease in the percentage of diastolic blood pressure and glycated hemoglobin. Modulation of NO production and the expression of apolipoproteins	[29]
Single-arm, non-controlled, non-randomized, prospective pilot clinical trial with thirty hypercholesterolemic volunteers	Dietary supplementation with highly standardized phenols (100 mg olive extract, 4–9% HT/day for four weeks)	Modulation of total cholesterol, LDL, HDL, systolic blood pressure, pulse pressure, uric acid, and fasting plasma glucose	[30]
Multicenter, randomized, double-blind controlled study involving 158 patients with mild or moderate hypercholesterolemia	Treatment with food supplement “Body Lipid” preparation (5 mg HT/day for four weeks)	Lowering of LDL levels with a reduction of −26.3%	[31]
Randomized, double-blind, controlled, crossover trial involving eighty-four subjects with hypertriglyceridemia, men and women, aged 45–65 years	Treatment with supplement containing 3.3 mg of HT from an olive fruit extract (three capsules/day for twenty weeks)	Decrease in LDL and triglycerides in plasma	[32]
Randomized, double-blind, controlled, crossover trial involving eighty-four subjects with hypertriglyceridemia, men and women, aged 45–65 years	Treatment with supplement containing 9.9 mg of HT (three capsules/day for twenty weeks)	Increase in endothelial function and reduced ox-LDL. Decreased systolic and diastolic blood pressure	[33]
Randomized double-blinded, placebo-controlled crossover trial involving twenty-eight patients, aged 18–65 years	Capsules incorporating 15 mg HT/day for three weeks	Increase in oxidation biomarkers, total antioxidant status, and SOD1. Decrease in NO metabolites and MDA	[34]
Intervention in fourteen volunteers with mild hyperlipidemia	45 mg/day for eight weeks	No influence on biomarkers of CVD and inflammation, electrolyte balance, blood lipids, and liver or kidney functions. Increase in endogenous vitamin C concentration	[35]
Intervention in five 32–68 years old male patients	25 mg of HT the first day and 12.5 mg/day the following three days	Decrease (46%) in serum TXB2 production. Enhancement of plasma antioxidant capacity. No effects on urinary 8-isoPGF2a and plasmatic albumin, bilirubin, uric acid, vitamin E, vitamin A, and β-carotene	[36]
Randomized, double-blind placebo-controlled trial involving eighty adolescent patients with biopsy-proven NAFLD	Pharmaceutical capsules containing 3.75 mg of HT, two capsules/day for four months	Decrease in triglyceride levels, oxidative stress parameters, insulin resistance, and steatosis	[37]
Randomized, double-blind prospective design including twenty-nine women with overweight/obesity	HT in two different doses (15 and 5 mg/day) for six months	Weight and visceral fat mass loss after four weeks	[38]
Sixty adults with overweight/obesity and T2D mellitus	60 g of conventional whole wheat bread or whole wheat bread enriched with HT (54 mg/100 g)	HT-enriched bread group showed greater fat mass, glucose, blood lipid, insulin, TNF-α, adiponectin, and HbA1c decrease than whole wheat bread group	[39]

**Table 2:** Other EVOO phenolic compounds evaluated in animal models and human clinical studies against cardiovascular and metabolic diseases.

Compound Tested	Type of Intervention/Model	Concentration Tested	Effects	References
Animal studies				
Oleuropein (Olp)	Female New Zealand white rabbits	0.4 mg/kg b.w. daily for six weeks	Increase in the capacity of LDL to counteract oxidation and reduction in plasmatic levels of total, esterified, and free cholesterol	[40]
	Male healthy or hypercholesterolemic New Zealand white rabbits	10–20 mg/kg b.w. daily for six weeks	Reduction in infarct extent. Decrease in protein carbonyl and plasma lipid peroxidation product concentrations Reduction in total cholesterol and triglyceride concentrations in plasma	[41]
	Male New Zealand white rabbits preconditioned with ischemia/reperfusion	20 mg/kg b.w. daily for six weeks	Increase in PI3K/Akt, eNOS, AMPK, and STAT 3 phosphorylation	[42]
	Adult male Lewis rats with experimental autoimmune myocarditis	20 mg/kg b.w. daily for four weeks	Limitation of left ventricular end-systolic diameters, left ventricular end-diastolic diameters, left ventricular end-diastolic pressures, and improvement of ejection fractions. Inhibition of macrophage, CD4+, and CD8+ infiltration in myocardium. Decrease in serum IL-1β, TNF-α, and IL-6. Reduction in myocardial levels of p-IκBα, IKKα, and NF-κB p65	[43]
	Experimental ischemia/reperfusion (myocardial I/R) in adult male Sprague-Dawley rats	20 mg/kg b.w. daily for two days	Inhibition of myocardial infarction extent, LDH and CK MB serum levels, and attenuation of p53, ERK, caspase 3, MEK, and p-IκBα protein expression. Decrease in MDA, IL-1β, IL 6, and TNF-α; increase in GSH, SOD, and catalase levels	[44]
	Alloxan-induced diabetic male New Zealand rabbits	20 mg/kg b.w. for sixteen weeks	Reduction in oxidative stress and hyperglycemia. Restoration of MDA levels, blood glucose, and most of the non-enzymatic and enzymatic antioxidant defenses	[45]
	Male TSOD mice	Olp-containing formulation “OPIACE”, Olp content exceeding 35% (*w*/*w*) for twenty-four weeks	Attenuation of impaired glucose tolerance and hyperglycemia from 10 to 24 weeks of age. No effects on obesity. Mild reduction in plasmatic oxidative stress by 26.2%	[46]
	HFD five-week-old male C57BL/6N mice	Olp-supplemented diet plus 0.03% (*w*/*w*) Olp	Reduction in body weight increment and visceral adiposity. Counteraction of elevations of adipogenic-related gene expression involved in WNT10b- and galanin-mediated signaling in adipose tissue	[47]
	Eight-week-old male normotensive Wistar-Kyoto (WKY) rats or spontaneously hypertensive rats (SHR)	2 mL saline or oral gavage of Olp (60 mg/kg/day)	Significantly lowered blood pressure and modulation of Nrf-2 at hypothalamic level	[52]
Oleacein (Ole)	HFD male C57BL/6JOlaHsd mice	20 mg/kg b.w. daily for five weeks	Prevention of the enhancement of adipocyte size and reduction in the inflammatory infiltration of lymphocytes and macrophages in adipose tissue. Restoration of the expression of PPARγ, FAS, adiponectin, SREBP-1, and Glut-4	[48]
	HFD male C57BL/6JolaHsd mice	20 mg/kg b.w. daily for five weeks	Down-regulation of plasma glucose, cholesterol, and triglyceride serum levels in comparison with normocaloric diet-treated mice. Modulation of FAS and SREBP-1	[49]
	12-week-old female BALB/c pristane-treated mice	Ole-enriched diet (0.01% (*w*/*w*)) for 24 weeks	Normalization of eNOS synthase and NADPH oxidase-1 overexpression	[50]
Tyrosol (Tyr)	LFD or HFD male C57BL/6J mice	LFD, HFD, or HFD supplemented with 0.025% (*w*/*w*) Tyr for 16 weeks	Binding with PPARα and activation of the transcription of downstream genes leading to lowered body weight and hepatic lipid accumulation	[51]
Human clinical studies				
Oleuropein (Olp)	Six healthy volunteers (males, non-smokers, aged 27–33)	12.6, 23.7, 33.7, and 39.5 mg conveyed through olive oil, four times after one month of washout	Decrease in urinary excretion of 8-iso-PGF2a,	[54]
	Randomized, double-blinded, placebo-controlled crossover study involving forty-six middle-aged overweight men	Four capsules as a single dose (51.1 mg), daily for twelve weeks	28% increase in pancreatic β-cell responsiveness and 15% increase in insulin sensitivity. Increase in IGFBP-1, IGFBP-2, and IL-6 concentrations. No effects on lipid profile, blood pressure, body composition, ultra-sensitive CRP, TNF-α, IL-8, carotid intima-media thickness, and liver function	[58]
Tyrosol (Tyr)	Thirty-three volunteers (twelve women, twenty-one men) aged 50–80 with at least three major CVD risk factors	1.4 + 25 mg Tyr + 0.2 HT (female volunteers) or 2.8 + 50 mg Tyr + 0.4 mg HT (male volunteers) conveyed through white wine, daily for four weeks	Lowering of three circulating ceramide (Cer) ratio levels and the alterations in plasma diacylglycerols concentrations	[55]
Oleocanthal (Olc)	Randomized, single-blinded crossover study, involving ten T2D patients	120 g white bread combined with 39 g butter, 39 g butter, and 400 mg ibuprofen, 40 mL OO (phenolic content < 10 mg/Kg), 40 mL OO with 250 mg/Kg Olc or 40 mL OO with 500 mg/Kg Olc	Postprandial dose-dependent reduction in platelets’ sensitivity	[60]
	Double-blind, randomized, controlled crossover study involving nine healthy men	40 mL of EVOO matched for total phenolic content, either Tyr-poor with 1:2 Ole/Olc or 2:1 Ole/Olc, or predominantly Tyr	Inhibition of collagen-stimulated platelet aggregation consumption of EVOO rich in Olc. Enrichment with Ole and Tyr did not lead to significant changes	[61]
	Randomized, double-blind, crossover trial performed on people aged 40–65 years with obesity and prediabetes	EVOO rich in Ole and Olc or OO for 1 month	Decrease in weight, BMI, and blood glucose and increase in antioxidant defenses after treatment with EVOO in comparison with OO group	[62]

**Table 3:** Effects of polyphenol-enriched EVOO in human clinical studies against cardiovascular and metabolic diseases.

Type of Intervention	Concentration Tested	Effects	References
Clinical studies			
EUROLIVE study: crossover, controlled trial involving 200 healthy male volunteers aged 20–60	3-week sequences of 25 mL/day of three OO with low (2.7 mg/kg), medium (164 mg/kg), and high (366 mg/kg) phenolic content	Counteraction of LDL oxidation with a significant decrease in IL8RA, ADRB2, OLR1, and CD40 gene expression and a decrease in LDL oxidation and intercellular adhesion molecule 1. Decrease in plasma apo B-100 concentrations and the number of small and total LDL particles	[65,66,67,68]
Crossover study design involving twelve male volunteers aged 20 to 22	40 mL of OO with low (2.7 mg/kg), moderate (164 mg/kg), and high (366 mg/kg) phenolic content (single administration)	Decrease in the degree of LDL oxidation proportionally to the phenolic content of OO	[69]
OLIVAUS study: double-blind, crossover, randomized, controlled trial involving fifty healthy volunteers (seventeen men, thirty-three women) aged 18–75	60 mL/day of either low-polyphenol EVOO (86 mg/kg) or high-polyphenol EVOO (360 mg/kg) for three weeks	Decrease in peripheral and central systolic blood pressure and plasma ox-LDL. Increase in TAC only in the high-polyphenol EVOO consumption. Decrease in hs-CRP levels only in the high-polyphenol EVOO-treated group	[70,71,72,73].
Randomized, controlled, parallel-arm clinical trial involving forty men and post-menopausal women aged 20–75 with at least one classic CVD risk factor	Daily amount of 25 mL of ROO or EVOO with meals for 6 weeks	Reduction in plasma LDL-cholesterol and plasma CRP	[74]
Randomized, controlled, crossover trial involving thirteen pre-/hypertensive patients aged 20 to 75 (7 men and 6 women)	30 mL of two similar OOs with moderate- (289 mg/kg) or high- (961 mg/kg) polyphenol content (single administration)	Postprandial enhancement of PPARα-, PPARγ-, PPARδ-, (PPAR)BP-, CD3-, 6ATP-binding cassette transporter-A1 and scavenger receptor class B type 1 gene expression in white blood cells	[75]
Double-blind, randomized, crossover dietary intervention study involving twenty-four young women with stage 1 essential hypertension or high-normal blood pressure	30 mg/day of polyphenols from OO for four months	Decrease in diastolic and systolic blood pressure, associated with reduction in serum asymmetric dimethylarginine (ADMA), plasma CRP, and ox-LDL only through the polyphenol-rich OO diet. Increase in plasma NO metabolites and hyperemic area after ischemia	[76]
Randomized, controlled, crossover study involving thirteen pre/hypertensive patients aged 20 to 75 (7 men and 6 women)	30 mL of two similar OOs with moderate- (289 mg/kg) or high- (961 mg/kg) polyphenol content (single administration)	Improvement of endothelial function measured as ischemic reactive hyperemia, and decrease in ox-LDL in plasma after intake of EVOO with high-polyphenol content	[77]
Aragon Workers’ Health Study: prospective cohort study involving 2318 participants	Normal diet for four years	Lowering of the risk of CVD measured as presence of plaques in carotid and femoral arteries and coronary calcium associated with higher intake of polyphenols from EVOO	[78]
Randomized crossover and blind trial on fourteen healthy and fourteen male hypertriacylglycerolaemic subjects, aged 21–38	Diet supplemented with ROO with no polyphenols or tocopherols or EVOO containing 1125 mg polyphenols/kg and 350 mg tocopherols/kg, for one week	Hypertriacylglycerolaemic and healthy volunteers showed lower incremental area under the curve for sVCAM-1 and sICAM-1 after the EVOO intervention	[79]
Randomized, sequential crossover study involving twenty-one hypercholesterolemic volunteers (five men and sixteen women) aged 53 to 68	Breakfasts including 40 mL VOO with either a low (80 ppm) or high (400 ppm) polyphenolic content	Higher decrease in plasminogen activator inhibitor-1 PAI-1 activity in plasma 2 h following the high-phenol meal than after the low-phenol meal	[80]
Randomized, parallel, controlled clinical trial in ninety healthy subjects aged 20 to 50	Mediterranean diet with washed virgin olive oil (WOO, 55 mg/kg) or VOO (328 mg/kg) for three months	Down-regulation of proatherogenic genes in peripheral blood mononuclear cells, and decrease in plasma inflammatory and oxidative status and the expression of related genes	[81]
Randomized, crossover, and controlled study involving fifty-one healthy adults	30 mL per day of standard VOO (124 ppm of phenolic compounds and 86 ppm of triterpenes), functional OO (487 ppm of phenolic compounds and enriched with 389 ppm of triterpenes), or optimized VOO (490 ppm of phenolic compounds and 86 ppm of triterpenes) for three weeks	Plasmatic reduction in oxidized lipids, inflammatory biomarkers (TNF-α and other cytokines), indicators of DNA damage and vascular damage	[84]
Randomized, controlled, double-blind crossover trial involving twenty adults (mean age 56.1 years; ten women, ten men) at risk for T2D	50 mL of ROO without polyphenols or high-polyphenolic EVOO (single administration)	Improvement of endothelial function assessed as flow-mediated dilatation. No significant activity on diastolic or systolic blood pressure	[85]
Eleven overweight T2D patients	First four weeks (wash-out period) administration of ROO (polyphenols not detectable), second four weeks with EVOO (25 mL/day, 577 mg of phenolic compounds/kg)	Reduction in fasting plasma HbA1c levels and glucose as well as body weight, BMI, and serum levels of ALT and AST	[86]
